# Adding NBPT to urea increases N use efficiency of maize and decreases the abundance of N-cycling soil microbes under reduced fertilizer-N rate on the North China Plain

**DOI:** 10.1371/journal.pone.0240925

**Published:** 2020-10-28

**Authors:** Gaoyuan Liu, Zhanping Yang, Jun Du, Ailing He, Huanhuan Yang, Guangyuan Xue, Congwen Yu, Yuting Zhang

**Affiliations:** 1 Institute of Plant Nutrition and Environmental Resources, Henan Academy of Agricultural Sciences, Zhengzhou, China; 2 Institute of Agricultural Research Center, Pingdingshan Academy of Agricultural Sciences, Pingdingshan, China; University of Queensland, AUSTRALIA

## Abstract

Urease inhibitor (UI) and nitrification inhibitor (NI) can reduce N losses from agricultural soils but effects of inhibitors on N cycle are unclear. A field experiment was conducted with maize to test effects of UI (N-(n-Butyl) thiophosphoric, NBPT) and NI (3,4-dimethylepyrazolephosphate, DMPP) on N uptake and N-cycling soil microbes. Five treatments were imposed: no N fertilizer input (CK), conventional fertilization (CF) and 80% of urea input with NBPT (80%U+UI), with DMPP (80%U+NI) and with half NBPT and half DMPP (80%U+1/2(UI+NI)). There were no significant differences in biomass between 80%U+UI, 80%U+NI and CF but harvest index was increased under 80%U+UI and 80%U+NI. Compared to CF, N use efficiency of grain under 80%U+UI was increased by 7.1%, whereas grain yield and N uptake under 80%U+1/2(UI+NI) were decreased by 8.2% and 9.4%, respectively. The peak soil ${\mathrm{NO}}_{3}^{‐}‐N$ content was at about 15 days after fertilization (DAF) under CF but 30 DAF under the inhibitor treatments. In soils of 80%U+UI, the activities of urease and nitrate reductase were decreased between 15–45 DAF and between 5–30 DAF. The abundance of N-cycling soil microbes was affected: 80%U+UI and 80%U+NI reduced the copies of the *amoA* AOA and *nir* genes at about 15 days and reduced the copies of the *amoA* AOB gene at about 30 days. Correlation analysis indicated that there were significant positive relationships between *amoA* AOB gene and ${\mathrm{NH}}_{4}^{+}‐N$, as well as between *nirK* gene and ${\mathrm{NO}}_{3}^{‐}‐N$. Overall, urea applied with NBPT has greater potential for improving maize N use efficiency and inhibiting nitrification under reduced fertilizer-N applications.

## Introduction

Worldwide demand for N fertilizer is predicted to increase at a rate of approximately 1.6 Tg yr^-1^. About half of the increase is expected to be in China (18%), India (17%) and Latin America (18%) [1]. Urea (CO[NH_2_]_2_) is the dominant form of N fertilizer used in Chinese agricultural soil as well as the world, because of its relative ease of transport and application [2, 3]. With surface applications of urea, N gaseous losses account for more than 40%-50% of the N applied under some environmental and edaphic conditions [4]. The processes of nitrification and denitrification are considered as the two major microbial ${\mathrm{NH}}_{4}^{+}‐N$ transformation pathways and also the main pathway of N loss from soil [5].

Nitrification inhibitor (NI) retards nitrification by deactivating ammonia oxidation [6–8]. Ammonia oxidation, a rate-limiting step in nitrification, catalyzed by the *amoA* gene encoding ammonia monooxygenase is carried out by ammonia-oxidizing bacteria (AOB) and ammonia-oxidizing archaea (AOA), which play important role in nitrification-derived N_2_O [9]. NI can decrease the abundance of *amoA* genes [8], N_2_O emissions and NH_3_ volatilization [10] in soils. Urease inhibitor (UI) delays urea hydrolysis and consequently affects the processes of nitrification and denitrification [11]. In denitrification, nitrite reductase is encoded by the *nirK* and *nirS* genes and has been used as a molecular marker for denitrifying bacteria [12]. UI or/and NI are added to urea, which can improve crop N use efficiency (NUE) and N uptake. When UI combines with urea, its solubility and diffusivity values are similar to those of urea. This facilitates its inhibitory effects on urea hydrolysis by urease [13, 14]. For NI, it slows the first step of NH_3_ oxidation and reduces NH_3_ conversion to ${\mathrm{NO}}_{3}^{‐}‐N$ and thus the extent of soil leaching [15]. It is well known that plant N uptake generally depends on the soil ${\mathrm{NO}}_{3}^{‐}‐N$, because ${\mathrm{NH}}_{4}^{+}‐N$ is rapidly converted to ${\mathrm{NO}}_{3}^{‐}‐N$ through nitrification followed by denitrification. However, there are few reports that focus on the effects of inhibitors on the dynamic changes in soil ${\mathrm{NO}}_{3}^{‐}‐N$, not on the nitrifiers/denitrifiers. Additionally, there is also debate regarding the economic benefits of inhibitors because some studies in which yield increases are not always observed, despite the additional cost [16–18].

Maize (*Zea mays* L.) is an important oil and cash crop and China contributes nearly 25% of global production [19]. Meanwhile, around 30% of Chinese maize production is on the North China Plain (NCP) [20]. Simultaneous fertilization and sowing are key agricultural practice in maize planting on the NCP, as later topdressing is very difficult, especially after tasseling (VT stage). Overfertilization with N at sowing increases N loss and so leads to N deficiency after tasseling, such as has been found in some studies where the maize N recovery rate was less than 40% [21, 22]. Meanwhile, overuse N fertilizer application increases the risk of nitrate accumulation and leaching of soil, which leads to groundwater contamination. A survey of nitrate concentration of 1,139 groundwater samples on the NCP, Zhao et al. [23] reported that the average nitrate value was 11.9 mg L^-1^, with 34.1% of samples exceeding the WHO standard due to overuse N fertilizer. Undoubtedly, this worrying result is associated with the overuse N fertilizer in the sampled area.

The objectives of this study were (ⅰ) to examine the effects of UI or/and NI on maize yield, N uptake and NUE under reduced fertilizer-N applications, (ⅱ) to compare the dynamic changes in ${\mathrm{NO}}_{3}^{‐}‐N$ contents, enzyme activities and N-cycling genes in soils. It is hoped this study will provide optimal fertilization regime for increasing maize yield and improving soil environment under reduced fertilizer-N applications.

## Materials and methods

### Site description

Our experiment site was located in Gucheng town (34°12’ N, 113°33’ E) of Yuzhou city, Henan province on the NCP. The field study was carried out on private land with the permission of the land owner and did not involve endangered or protected species. This region has a temperate, semi-humid monsoon climate, where the highest temperature and most of rainfall simultaneously occur in summer. Annual precipitation is 673 mm, of which 70% occurs in June-August and annual evaporation is 946 mm. Mean annual temperature is 14.4°C and the frost-free period is 214 days. The soil taxonomy of the experiment site is an Aridic Haplustepts, which belongs to Inceptisols in USDA classification system. Before this study, the soil properties in the 0–20 cm depth band were: organic C 8.6 g kg^-1^, total N 0.6 g kg^-1^, available P 5.24 mg kg^-1^, available K 121.4 mg kg^-1^ and pH 7.94.

### Experiment design

A field experiment had five treatments: (ⅰ) no N fertilizer input (CK), (ⅱ) conventional fertilization (CF) and (ⅲ) 80% of urea input with NBPT (80%U+UI), (ⅳ) with DMPP (80%U+NI) and (ⅴ) with half NBPT and half DMPP (80%U+1/2(UI+NI)). The area of each plot was about 45 m^2^ (5.6 m × 8.0 m), and plots were completely randomized with respect to treatment and each treatment was repeated three times. The plot distribution, buffer area and guard rows are illustrated in S1 Fig. The application levels of fertilizers and inhibitors are listed in Table 1. Fertilizer application and sowing were carried out simultaneously using an integrated machine. No topdressing fertilizers were applied in any treatments. The maize cultivar was Zhengdan 1002, and it was sown in Jun 2019 at a density of 67500 plants hm^-2^ and a row spacing of 60 cm. All rows in each plot were harvested in late September 2019. Field managements were consistent with local practice for the control of pests, diseases and weeds, and for irrigation.

**Table 1:** Application levels of fertilizers and inhibitors.

Treatment	Fertilizer (kg·km^-2^)			Inhibitor
	N	P_2_O_5_	K_2_O	
CK	0	60	60	/
CF	225	60	60	/
80%U+UI	180	60	60	1.5% of urea dosage
80%U+NI	180	60	60	1.5% of urea dosage
80%U+1/2(UI+NI)	180	60	60	0.75% of urea dosage

### Sampling

At 5, 15, 30, 45, 60 and 90 days after fertilization (DAF), three soil samples were taken with an auger from each plot, respectively. Each sample was split into three subsamples from soil depths of 0–20, 21–40 and 41–60 cm. Samples were combined between replicate treatments to give a bulk sample at the same depth band and immediately stored in a cooler at 4°C to determine ${\mathrm{NO}}_{3}^{‐}‐N$ content. In laboratory, subsamples (50 g) from the 0–20 cm depth band were separated and stored at 4°C for analysis of enzyme activity. Meanwhile, some of subsamples at 15, 30 and 45 DAF from the 0–20 cm depth band were stored at -20°C used for later analysis of microbial abundance and the rest were air-dried for analysis of physiochemical property. The grain and straw from each plot were collected separately by hand to determine aboveground biomass. At harvest, 500 g samples of air-dried grain and subsamples (10 plants) of air-dried straw were taken from each plot and carried to the laboratory to determine N uptake and NUE.

### Sample analysis

The mass yields of grain and straw were determined at the moisture levels of 12% and 11%, respectively. Harvest index was obtained by the ratio of grain yield to biomass. The N contents of grain and straw were measured by the method of concentrated H_2_SO_4_ digestion. The NUE was calculated as:

$$NUE\;(\%)=\frac{N_{1}-N_{2}}{N_{i}}\times 100\%$$

where *N*_1_ and *N*_2_ are the N uptake under N and no-N fertilizer treatment (kg hm^-2^), respectively, and *N*_*i*_ is net N input from fertilizer (kg hm^-2^).

The analyses of organic C, total N, ${\mathrm{NH}}_{4}^{+}‐N$, available P, available K and moisture in soils were according to the methods described by Lu [24]. The ${\mathrm{NO}}_{3}^{‐}‐N$ content was determined using a continuous flow analyzer (CleverChem 200, DeChem-Tech, GmbH Corporation, German) after being extracted with 2M KCl [25]. Enzyme activities were determined by the methods described by Hu et al. [26]. For urease, the samples were incubated with 1 mL methylbenzene, 10 mL of 10% urea solution and 20 mL of citrate buffer at 30°C for 24 h, ${\mathrm{NH}}_{4}^{+}‐N$ concentration in the mixture was determined using an UV-visible spectrophotometer (UV-2600, SHIMADZU, Tokyo). For nitrate reductase, the samples were incubated with 1 mL of 0.8 mM 2,4-dinitrophenol solution, 1 mL of 0.05% KNO_3_ solution, 1 mL of 1% glucose solution and 7 mL of anaerobic deionized water at 30°C for 24 h, ${\mathrm{NO}}_{2}^{‐}‐N$ concentration in the mixture was determined using the UV-visible spectrophotometer.

Total DNA was extracted using an E. Z. N. A. Soil DNA Kit (Omega Bio-tek, Norcross, GA, USA) according to the manufacturer’s instructions. Extracted DNA were stored at -20°C until used in downstream analyses. The quantity and quality of the extracted DNA were determined using a Nanodrop Spectrophotometer (Nanodrop 2000, Thermo Fisher Scientific, USA). The primers of the Arch-amoAF:Arch-amoAR (*amoA* AOA), amoA-1F:amoA-2R (*amoA* AOB), nirSCd3Af:nirSR3cd and nirKF1aCu:nirKR3Cu were used to amplify the *amoA* AOA, *amoA* AOB, *nirS* and *nirK* gene fragments, respectively [27–29]. All gene quantitative PCR (qPCR) amplifications were performed with a Rotor-Gene^®^ Q (CFX96Touch, BIORAD, USA) in 20 μL of the reaction mixture containing 10 μL of 2 × SYBR Green Master Mix (BioTeke, Beijing), 0.4 μM of forward and reverse primer, 1 μL of template DNA and 8.2 mL of sterile distilled water. Target genes were quantified using the primers and qPCR programs [30].

### Statistical analysis

The differences in biomass between treatments were tested with a one-way ANOVA in SPSS v. 25.0, as well as N uptake, NUE, harvest index and gene copies. An LSD analysis was conducted to compare the difference in ${\mathrm{NO}}_{3}^{‐}‐N$ content and in enzyme activity in SAS v. 9.4. The Spearman’s coefficient and *p*-value were performed to quantify correlations between enzyme activities, N-cycling genes and physiochemical parameters using functions in psych package of R v. 3.6.0, and the corrplot package in R was used to display the results.

## Results

### Maize N uptake, N use efficiency and harvest index

Maize N uptake, NUE and harvest index were analyzed under different fertilization treatments (Table 2). Results showed that the differences in biomass between 80%U+UI, 80%U+NI and CF were not significant but that under 80%U+1/2(UI+NI) was significantly lower than under CF (grain 8.2% and straw 17.0%). Meanwhile, grain N uptake under 80%U+1/2(UI+NI) was significantly decreased by 9.4% than that under CF. Compared to CF, straw N uptake under the inhibitor treatments was decreased by between 18.9% and 31.3%. The grain NUE under 80%U+UI was significantly increased by 7.1% than that under CF, whereas the differences were not significant between 80%U+NI, 80%U+1/2(UI+NI) and CF. For harvest index, it was significantly increased under 80%U+UI and 80%U+NI compared to CF, but not under 80%U+1/2(UI+NI).

**Table 2:** Maize N uptake, NUE and harvest index under different fertilization treatments.

Treatment	Biomass (kg hm^-2^)		N uptake (kg hm^-2^)		Grain NUE (%)	Harvest index
	Grain	Straw	Grain	Straw		
CK	7639±204 c	6472±192 c	77.9±3.4 c	5.8±1.8 d	/	0.53±0.02 a
CF	10090±333 a	10856±478 a	124.4±7.1 a	40.5±4.4 a	18.2±1.9 b	0.48±0.02 b
80%U+UI	10394±598 a	10084±535 ab	123.5±5.9 a	32.9±2.8 b	25.3±2.6 a	0.51±0.01 a
80%U+NI	10265±224 a	9919±561 ab	119.3±8.2 ab	31.8±3.6 bc	23.0±2.8 ab	0.51±0.00 a
80%U+1/2(UI+NI)	9265±369 b	9008±348 b	112.8±4.0 b	27.8±1.9 c	19.4±1.4 b	0.50±0.01 ab

Values are means+SD (n = 3). Values in the same column followed by different lowercase letters are significantly different (*p* < 0.05). CK, no N fertilizer input; CF, conventional fertilization; 80%U+UI, 80%U+NI and 80%U+1/2(UI+NI) indicate 80% of urea input with NBPT, with DMPP and with half NBPT and half DMPP, respectively.

### Dynamic change of soil ${\mathrm{NO}}_{3}^{‐}‐N$ content

**Fig 1:**
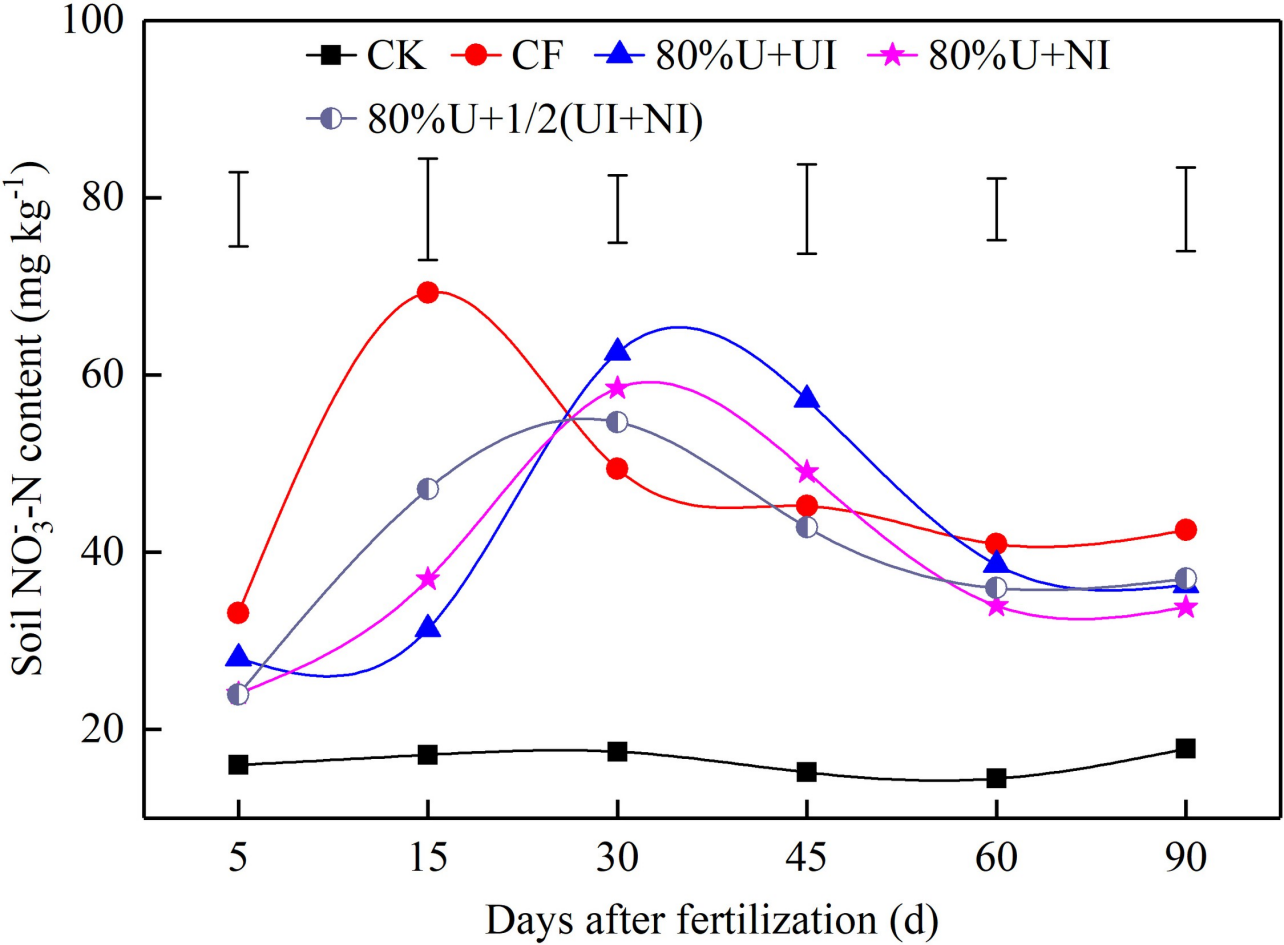
Mean ${\mathrm{NO}}_{3}^{‐}‐N$ content in the 0–60 cm soil depth band from 5 to 90 days after fertilization. CK, no N fertilizer input; CF, conventional fertilization; 80%U+UI, 80%U+NI and 80%U+1/2(UI+NI) indicate 80% of urea input with NBPT, with DMPP and with half NBPT and half DMPP, respectively. Vertical bars represent LSD_0.05_ between treatments.

In the 0–60 cm soil depth band, the mean ${\mathrm{NO}}_{3}^{‐}‐N$ content for each treatment was calculated from 5 to 90 DAF (Fig 1). Under CF, the peak ${\mathrm{NO}}_{3}^{‐}‐N$ content appeared at about 15 DAF, when it was significantly increased by 121.2%, 92.5% and 36.7% than under 80%U+UI, 80%U+NI and 80%U+1/2(NI+UI), respectively. With additions of the inhibitors, the peaks of ${\mathrm{NO}}_{3}^{‐}‐N$ content appeared at about 30 DAF. Those peaks were obviously increased compare to CF, with a range of 10.4%-26.6%. At 45 DAF, the ${\mathrm{NO}}_{3}^{‐}‐N$ content under 80%U+UI was still higher (30.9%, *p* < 0.05) than under CF. During the period 60 to 90 DAF, the differences in ${\mathrm{NO}}_{3}^{‐}‐N$ contents between N fertilizer treatments were not significant.

### Activities of urease and nitrate reductase

**Fig 2:**
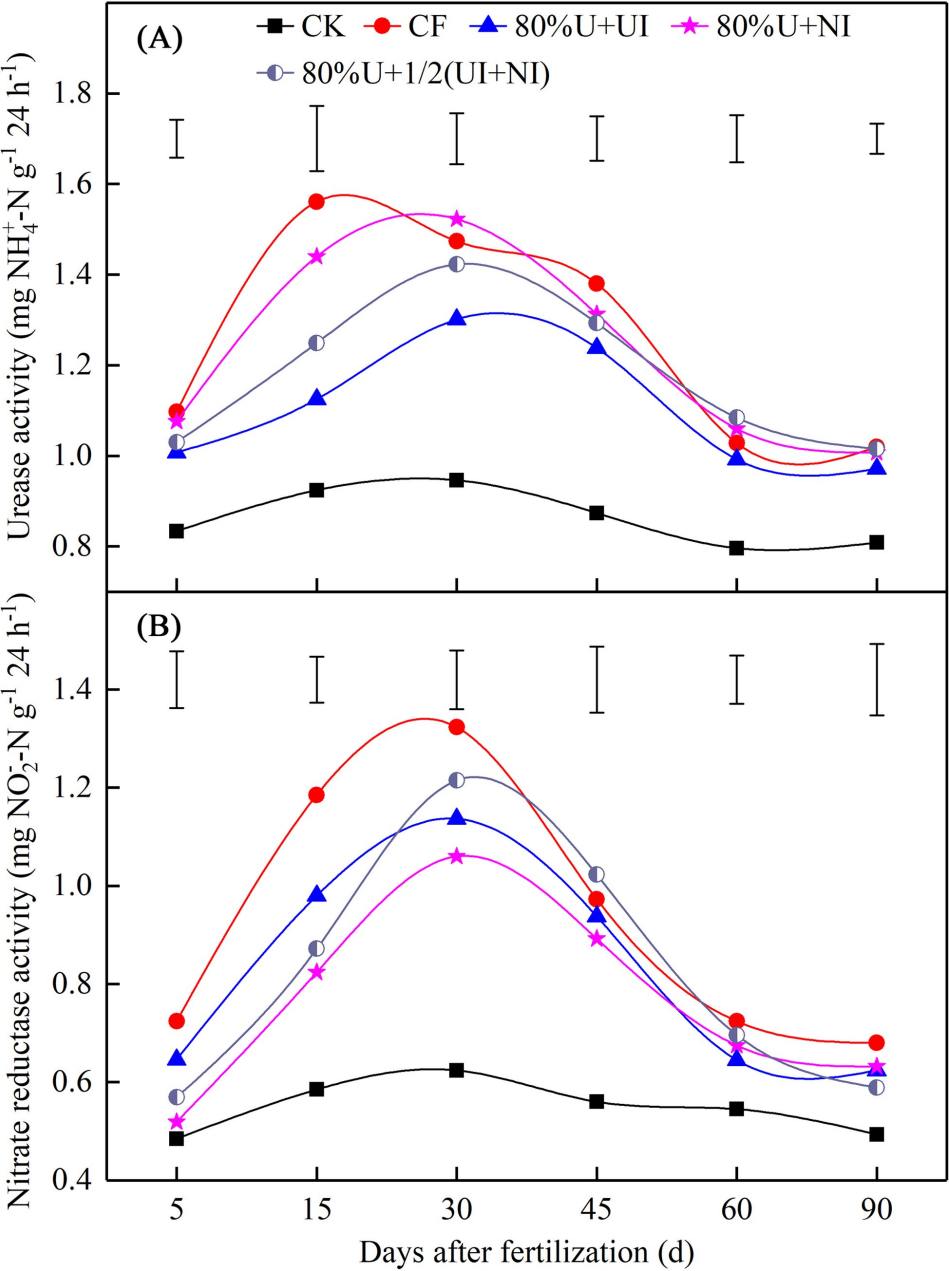
The activities of urease (A) and nitrate reductase (B) under different fertilization treatments. CK, no N fertilizer input; CF, conventional fertilization; 80%U+UI, 80%U+NI and 80%U+1/2(UI+NI) indicate 80% of urea input with NBPT, with DMPP and with half NBPT and half DMPP, respectively. Vertical bars represent LSD_0.05_ between treatments.

Compared to CF, the urease activity under 80%U+UI was significantly decreased by 17.3% between 15–45 DAF, with a range of 10.2%-27.9%. However, over the period 30–90 DAF, no significant differences were observed between 80%U+NI, 80%U+1/2(UI+NI) and CF (Fig 2A). The nitrate reductase activities under the inhibitor treatments were substantially decreased from 5 to 30 DAF compared to CF, and the reduction rate was between 18.6% and 27.1% (Fig 2B). During the period 45 to 90 DAF, the nitrate reductase activities under different treatments showed a downward trend but no significant differences were found between N fertilizer treatments (Fig 2B).

### Abundance of N-cycling genes

**Fig 3:**
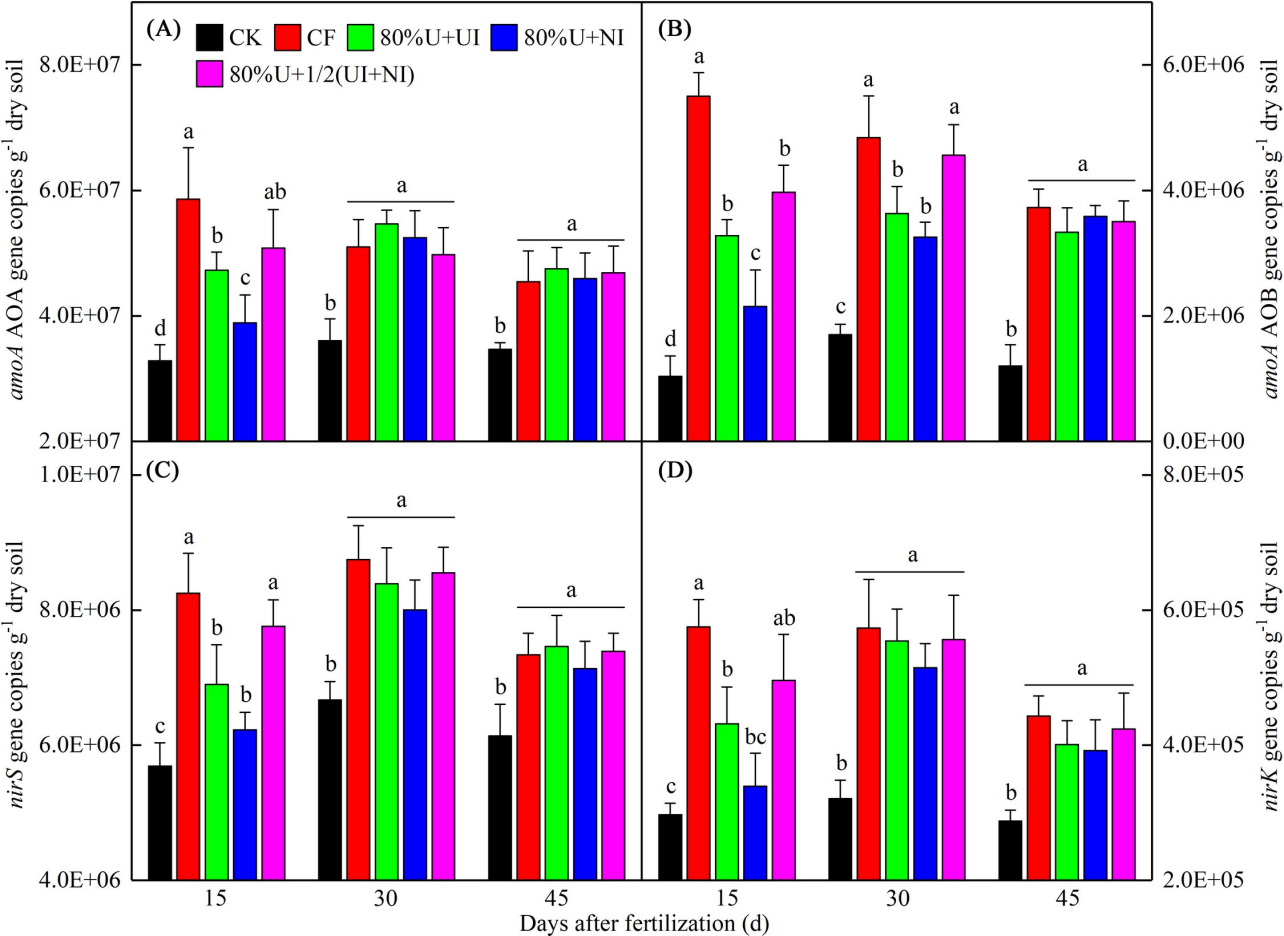
The N-cycling gene copies under different fertilization treatments. CK, no N fertilizer input; CF, conventional fertilization; 80%U+UI, 80%U+NI and 80%U+1/2(UI+NI) indicate 80% of urea input with NBPT, with DMPP and with half NBPT and half DMPP, respectively. Different lowercase letters above the bars represent significant differences (*p <* 0.05) between treatments.

The abundance of N-cycling genes was markedly affected by inhibitors, especially when UI or NI was used on their own (Fig 3). Compared to CF, the *amoA* AOA gene copies under 80%U+UI and 80%U+NI were significantly decreased by 22.3% and 40.1% at 15 DAF, respectively (Fig 3A). Meanwhile, the *amoA* AOB gene copies under 80%U+UI and 80%U+NI were significantly decreased by 44.5% and 65.6% at 15 DAF and by 36.8% and 29.3% at 30 DAF, respectively (Fig 3B). No significant differences in *amoA* genes were observed between 80%U+1/2(UI+NI) and CF from 30 to 45 DAF (Fig 3A and 3B).

Compared to CF, at 15 DAF, the copies of *nirS* and *nirK* genes were significantly decreased by 19.4% and 31.4% under 80%U+UI and by 28.7% and 49.2% under 80%U+NI, respectively (Fig 3C and 3D). However, no significant differences in *nir* genes were found between 80%U+1/2(UI+NI) and CF at 15 DAF. Additionally, the differences in *nir* genes between N fertilizer treatments were not significant at either 30 or 45 DAF (Fig 3C and 3D).

### Correlation analysis

**Fig 4:**
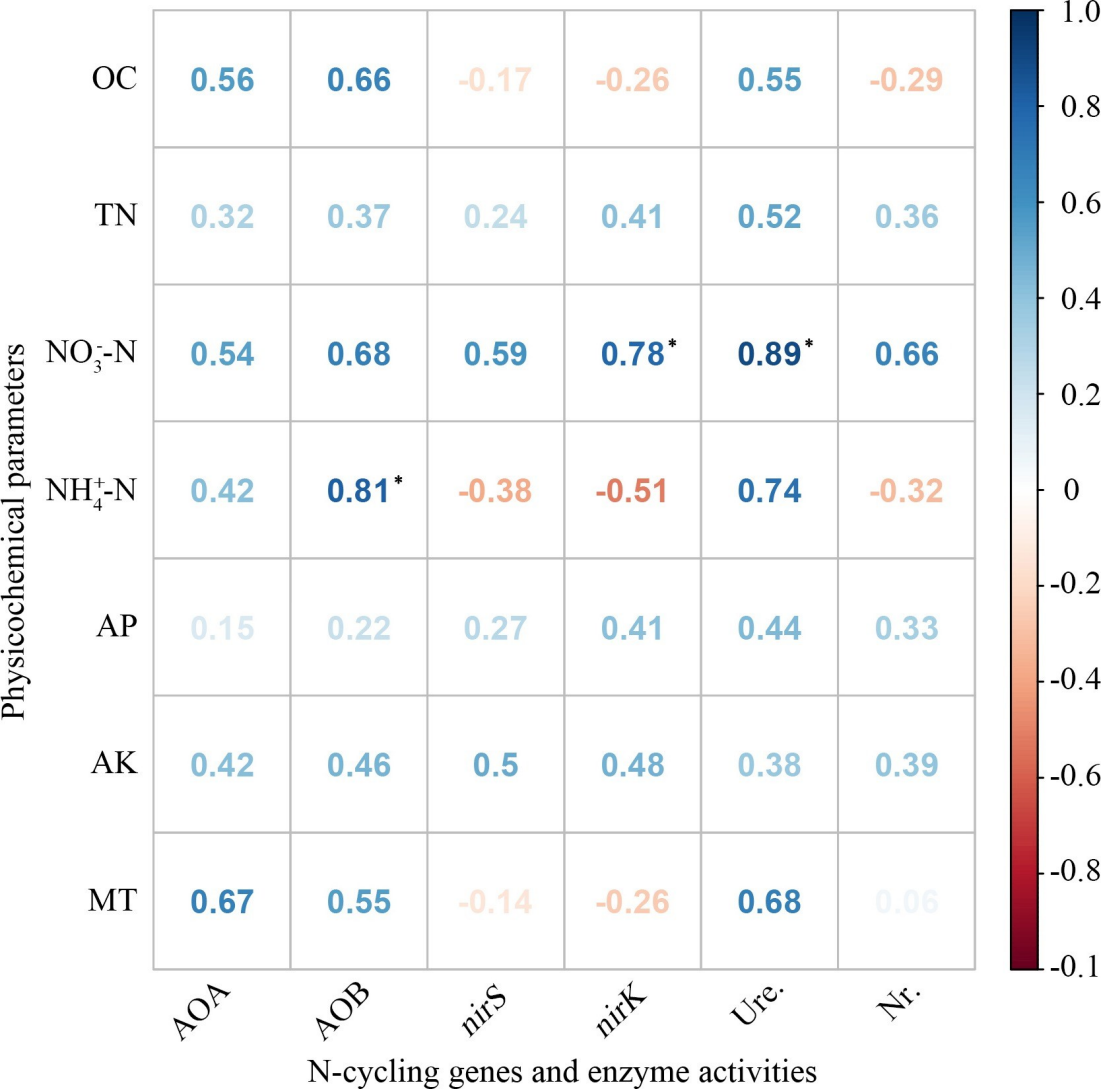
Correlation analysis between N-cycling genes, enzyme activities and physicochemical parameters in the soil. OC, organic C; TN, total N; AP and AK, available P and K; MT, moisture; AOA, *amoA* AOA; AOB, *amoA* AOB; Ure., urease activity; Nr., nitrate reductase activity. * represents significant correlation (*p* < 0.05).

The correlation analysis revealed significant relationships between enzyme activities, nitrifying/denitrifying genes and physiochemical parameters when different fertilization treatments were considered (Fig 4). There were significant positive relationships between *amoA* AOB gene and ${\mathrm{NH}}_{4}^{+}‐N$, as well as *nirK* gene and ${\mathrm{NO}}_{3}^{‐}‐N$. In contrast, the abundance of *amoA* AOA and *nirS* genes was relatively little affected by physiochemical properties. Additionally, there was a significant positive relationship between urease activity and ${\mathrm{NO}}_{3}^{‐}‐N$.

## Discussion

Using meta-analysis, Linquist et al. [31] have found that additions of the inhibitors led to an 8.0% increase in field crop N uptake. Similarly, our results indicate that under 80%U+UI and 80%U+NI, maize NUE was increased by 8.5% and 5.9%, respectively (Table 2). Previous studies also concluded that additions of the inhibitors improve maize yields in comparison to conventional urea alone [15, 32]. In present study, although the N fertilizer applications under 80%U+UI and 80%U+NI were reduced by 20%, their yields were not affected while harvest indexes were increased (Table 2). In other words, the addition of UI or NI increases maize yield and thus commercial return under reduced N fertilizer application. We also found that maize biomass and N uptake under 80%U+1/2(UI+NI) did not perform better than the addition of UI or NI alone. Also, these were obviously decreased compared to CF (Table 2). This finding has been described by Zaman et al. [11] and Frame [33] who attributed it to the addition of NI to urea with UI increasing NH_3_ volatilization in the soil.

Urea stabilized by inhibitor retards the N transformation process and extends the period over which N is available to the crop in the soil [34–36]. In our study, the ${\mathrm{NO}}_{3}^{‐}‐N$ contents under the inhibitor treatments were decreased at 15 DAF but then increased at 30 DAF (Fig 1). This result is consistent with that described by Azam et al. [5], who reported that when urea was applied with inhibitor, the enzyme activity and NH_3_ volatilization were reduced but the ${\mathrm{NO}}_{3}^{‐}‐N$ accumulation was increased. Moreover, our result showed that the ${\mathrm{NO}}_{3}^{‐}‐N$ content at 45 DAF under 80%U+UI was higher than that under CF (Fig 1)–this says urea applied with NBPT has a longer N supply period. This may be related to the reduction of urease activity at about 45 DAF (Fig 2A).

To some extent, UI retarded the urea hydrolysis by decreasing urease activity and so slowed the conversion of urea-N to ${\mathrm{NH}}_{4}^{+}‐N$, which led to a slow-down in the reduction of ${\mathrm{NO}}_{3}^{‐}‐N$ to ${\mathrm{NO}}_{2}^{‐}‐N$ [37, 38]. In our study, 80%U+UI decreased the urease activity between 15 and 45 DAF and decreased the nitrate reductase activity between 5 and 30 DAF (Fig 2). The inhibitory effect of 80%U+NI and 80%U+1/2(UI+NI) on urease activity was relatively weak in comparison to 80%U+UI (Fig 2A). The N-conversion related enzyme is the primary factor affecting inhibitory effect under dryland conditions [32]. Therefore, we speculate that urea applied along with NBPT has greater potential as an inhibitor of urea hydrolysis.

Fu et al. [38] reported that there were no significant differences in *amoA* AOA genes between urea only and with additions of the inhibitors in an acid red soil. In our study, the *amoA* AOA genes under 80%U+UI and 80%U+NI were decreased at about 15 DAF (Fig 3A). This result can be explained by differences in soil pH, inhibitor dosage and sampling times between our study and theirs. Our result showed that the *amoA* AOB genes under 80%U+UI and 80%U+NI were decreased between 15 and 30 DAF (Fig 3B). This is due to additions of the inhibitors which inhibited the growth of AOB [6]. Additionally, the inhibitory effect of 80%U+1/2(UI+NI) on the *amoA* genes was relatively week in comparison to UI or NI alone (Fig 3A and 3B). We speculate that this is related not only to inhibitor dosage, but also to the negative effect of NI addition to urea with UI [39]. The *nir* genes increase more with N fertilizer dosages in an alkaline soil [40]. Our result showed that the *nir* genes in the treatments with 80% fertilizer-N were obviously reduced at about 15 DAF, especially under 80%U+UI and 80%U+NI (Fig 3C and 3D). The results can be explained as that the increases in fertilizer-N provided sufficient substrate for denitrification but the additions of the inhibitor limited the conversion rate of ${\mathrm{NH}}_{4}^{+}‐N$ to ${\mathrm{NO}}_{3}^{‐}‐N$ [41, 42]. Additionally, we found that the inhibitor treatments did not affect the *nir* genes between 30 and 45 DAF (Fig 3C and 3D). This finding suggests that urea applied with NBPT and/or DMPP inhibits denitrification about 15 days, after that, the inhibitory effect has weakened, somewhat.

Nutrient availability, particularly of C and N, are the main factors affecting microbial abundance in the soil [43]. In our study, a significant positive relationship was observed between *amoA* AOB gene and ${\mathrm{NH}}_{4}^{+}‐N$ (Fig 4). This result has previously been confirmed by Chen et al. [44], who attributed it to added ${\mathrm{NH}}_{4}^{+}‐N$ to the soil which provided abundant N substrate and energy for the growth of AOB. Meanwhile, we found that there was a significant positive relationship between *nirK* gene and ${\mathrm{NO}}_{3}^{‐}‐N$ (Fig 4). It can be explained as that ${\mathrm{NO}}_{3}^{‐}‐N$ supplies a rich reaction substrate for the *nir* denitrifiers in the soil and so stimulates their reproduction, where the *nirK* denitrifiers are more sensitive to ${\mathrm{NO}}_{3}^{‐}‐N$ than the *nirS* denitrifiers [45].

## Conclusion

Our results show that, compared to conventional fertilization, the application of NBPT increased maize grain NUE and harvest index when the urea rate was reduced by 20%, although biomass and N uptake were not affected. With additions of the inhibitors, the peak ${\mathrm{NO}}_{3}^{‐}‐N$ contents in the 0–60 cm depth band were delayed by 15 days. The addition of NBPT to urea decreased the urease activity for about 45 days, which was longer than under the other treatments. The abundance of N-cycling soil microbes was affected by additions of NBPT or DMPP to the urea, decreasing the copies of the *amoA* AOA, *amoA* AOB and *nir* genes during the growth period. These results suggest that urea applied with NBPT has greater potential for improving maize NUE and inhibiting nitrification under reduced fertilizer-N applications on the NCP.

## Supporting information

S1 Fig The plot distribution, buffer area and guard row in this experiment. (TIF)
