# Supplementary figures and images for: Adding NBPT to urea increases N use efficiency of maize and decreases the abundance of N-cycling soil microbes under reduced fertilizer-N rate on the North China Plain
Source: PLoS One. 2020 Oct 28;15(10):e0240925. doi: 10.1371/journal.pone.0240925 (PMC7592763; doi:10.1371/journal.pone.0240925)

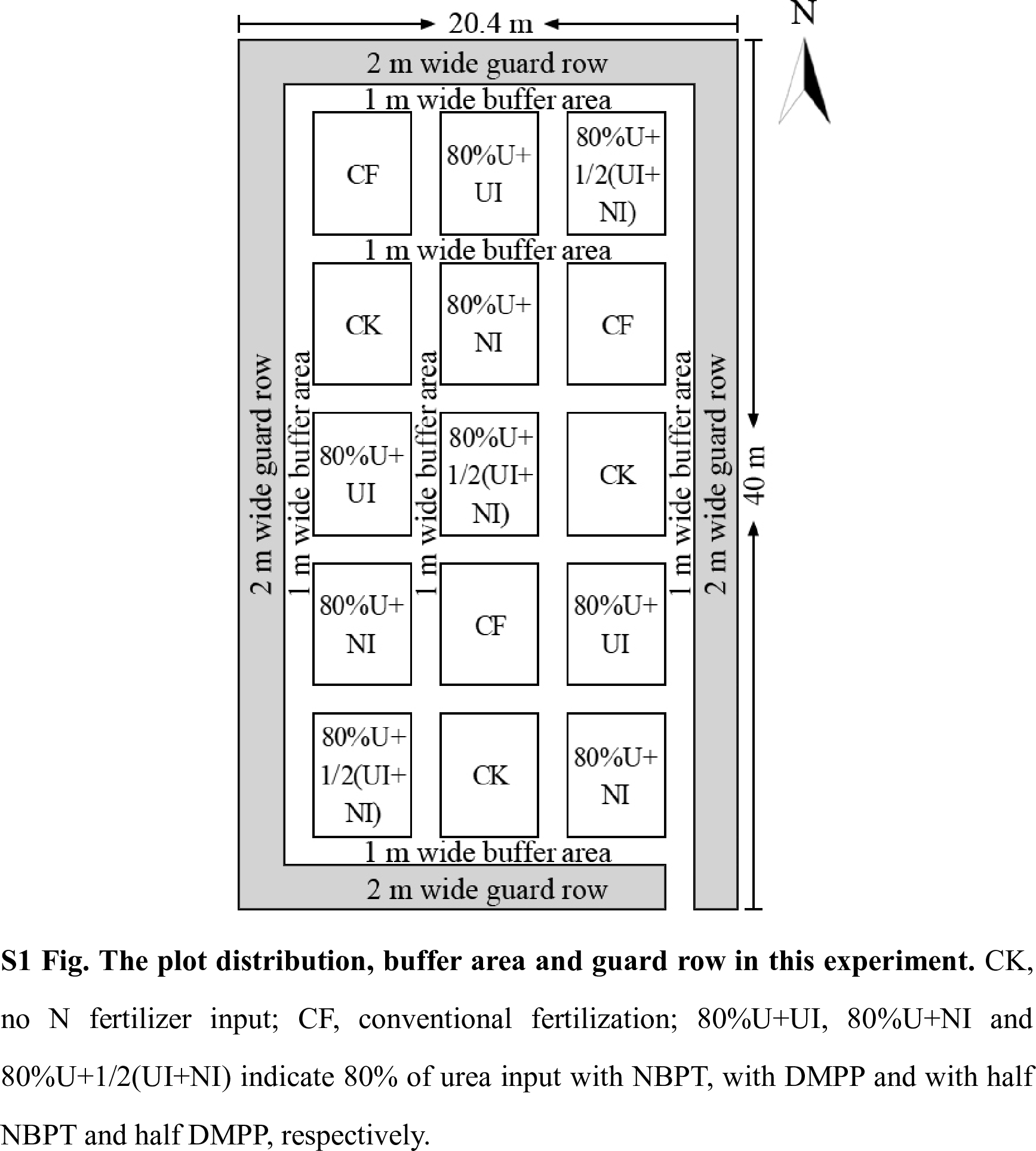

Supplement: S1 Fig — (TIF) [file pone.0240925.s001.tif]
